# Comparison of Two Posterior Chain Strength Training Protocols on Performance and Injury Incidence in Elite Youth Football Players

**DOI:** 10.3390/medicina62010140

**Published:** 2026-01-09

**Authors:** Manuele Ferrini, José Asian-Clemente, Gabriele Bagattini, Luis Suarez-Arrones

**Affiliations:** 1 Department of Sport Sciences, Universidad Pablo de Olavide, 41013 Sevilla, Spain; manuele.ferrini@gmail.com (M.F.); g.bagattini@me.com (G.B.); ljsuamor@upo.es (L.S.-A.); 2 FSI Lab, Football Science Institute, 18016 Granada, Spain; 3 Performance and Health Department, FC Lugano, 6900 Lugano, Switzerland

**Keywords:** eccentric hamstring strength, hamstring injury prevention, posterior-chain training, strength training, youth soccer players

## Abstract

*Background and Objectives*: This study compared the effects of two posterior-chain strength training strategies on eccentric hamstring strength, jump and sprint performance, and hamstring injury incidence in elite youth soccer players. *Materials and Methods*: Twenty-three players were randomly allocated to either a Nordic Hamstring Exercise Group (NHEG; *n* = 11) or a Deadlift + Leg Curl Slides Group (D + LCSG; *n* = 12). Both groups completed a 9-week in-season resistance training program consisting of one strength-oriented session (MD-4) and one power-oriented session (MD-2) per week, in addition to regular soccer training. Pre- and post-intervention assessments included eccentric hamstring strength (NordBord), countermovement jump (CMJ), and 10 m and 30 m linear sprint performance. *Results*: Eccentric hamstring strength increased significantly only in the NHEG (*p* ≤ 0.05), though this improvement did not transfer to enhancements in jump or sprint performance (*p* > 0.05). No significant changes were observed in the D + LCSG for any variable (*p* > 0.05), and no between-group differences were found across all performance outcomes. During the 12-week monitoring period, one hamstring injury was recorded, occurring in the NHEG. *Conclusions*: These findings suggest that, while the NHE elicited greater exercise-specific eccentric strength gains, neither posterior-chain strategy produced improvements in sprint or jump performance. However, given the small sample size and low number of injury events, these trends cannot be attributed with certainty to the implemented protocols, and both programs reported a low incidence of hamstring injuries per 1000 h of exposure with no statistically protective effect associated with the use of the NHE.

## 1. Introduction

Strength training is widely recognized as a fundamental component of modern football preparation [1], contributing to the development of neuromuscular qualities essential for high-intensity actions such as sprinting, jumping, shooting, and changes in direction [2]. In addition to performance enhancement, strength training may improve neuromuscular control and load tolerance, thereby minimizing the risk of lower-limb injuries [3]. Strength training thus contributes not only to improving performance but also to enhancing resilience and player availability [4]. The latter aspect is particularly important given that injuries represent a major concern in football at both amateur and elite levels, with significant implications for player health, team performance, and long-term career outcomes [5]. Of football-related injuries, hamstring strain injuries (HSIs) are among the most prevalent and recurrent injuries in men’s professional football [6]. HSIs account for a significant number of absence days, with substantial consequences for clubs, both financially and in terms of competitive performance [7,8]. The incidence of HSIs in men’s professional football has increased, from 12% in the 2001/2002 season to 24% in the 2021/2022 season, currently accounting for approximately 25% of all injuries [9]. Although the incidence of hamstring strain injuries is lower among youth players than in adults, youth football represents a particularly relevant population for investigation. HSIs remain the most common type of muscle injury in this population as well [10]. During adolescence, rapid changes in hormone release, body size, body composition [11], and neuromuscular control [12] make young football players particularly predisposed to this type of injury, which further underscores the importance of prevention at this stage [13]. Considering that injuries during adolescence may compromise the development and progression of young players, HSIs in youth players warrant particular attention [14]. Focusing on these injuries through preventive strategies is essential, not only to reduce injury risk but also to support sustained performance and resilience throughout a player’s career [15].

Currently, several methods have been proposed as potential strategies to reduce the risk of HSIs, with sprinting and high-speed running, in addition to eccentric-focused exercises, receiving the strongest consensus [16]. Other approaches, such as programs incorporating strength, balance, and stability exercises, have also been suggested to be effective in mitigating injury risk in adult football players [4,17,18]. Among youth athletes, exercise-based injury prevention programs have been shown to reduce injury rates by up to 46% [15]. Such programs typically combine multiple exercise modalities [19,20,21]. Among the different strategies adopted, the Nordic Hamstring Exercise (NHE) has gained prominence as an effective, evidence-based strategy for reducing hamstring injury risk [22]. Although several systematic reviews and meta-analyses report substantial reductions in HSI incidence of up to 51% following NHE implementation [22,23,24], these findings should be interpreted with caution. Many included studies compared the NHE with control conditions lacking structured preventive programs [25,26,27], basic flexibility routines [28], or non-elite populations [29], which may partly explain the magnitude of the reported effects. Consequently, the protective role of the NHE has been questioned [30], highlighting the need for direct comparisons with alternative posterior-chain exercises and for studies tracking actual injury incidence in elite football populations. To date, evidence from such head-to-head comparisons remains limited. Due to the logistical challenges associated with tracking injury incidence and the multifactorial nature of hamstring injuries, several studies have relied on indirect measures, such as muscle activation, as proxies for injury-preventive potential. For example, it has been suggested that greater biceps femoris activation during an exercise may indicate a higher capacity to reduce hamstring injury risk [31]. However, this assumption should be interpreted with caution, as muscle activation does not directly translate to injury prevention, and most studies adopting this approach have not assessed actual injury outcomes. Although the NHE has been shown to elicit higher biceps femoris activation compared with other exercises during eccentric actions [31], the extent to which these activation patterns are associated with a reduced incidence of hamstring injuries remains unclear. Other research compared sprint training with the NHE over four weeks, reporting promising adaptations in performance and putative risk factors [32]. More recently, a six-week study compared a hip-dominant eccentric exercise, the Romanian deadlift, with NHE (knee-dominant) regarding their effects on HSI risk factors [33]. The authors suggested that the Romanian deadlift may offer similar, or potentially even greater, benefits than the NHE for HSI prevention, targeting the biceps femoris muscle more proportionally [33]. Nevertheless, as these studies did not track actual injury incidence, their findings on preventive impact remain limited. Overall, the existing limitations in the current literature highlight the need for high-quality studies that track the impact of exercise strategies on injury incidence over longer follow-up periods.

Beyond injury prevention, sprint performance represents a key performance determinant in football and is a primary mechanism underlying hamstring strain injuries. The NHE has been widely implemented not only for its proposed protective effects, but also based on the assumption that increases in eccentric knee flexor strength may transfer to improved sprint performance [24,34]. Therefore, examining whether posterior-chain strength strategies translate into meaningful sprint adaptations is central to evaluating both the performance relevance and the preventive rationale of these interventions. Although this concept is theoretically appealing, particularly given the sprinting demands of football, certain aspects must be considered when comparing the NHE and sprinting. On the one hand, the NHE is a slow and linear movement performed from a kneeling position, whereas sprinting is a high-velocity, hip-dominant action involving substantial contributions from the gluteal muscles and proximal hamstrings [35]. Some authors have reported that the patterns of muscle activation between these two movements differ substantially [36]. These factors may explain why some studies found an inverse relationship between NHE strength and sprint times [37,38], while others showed no association [39,40,41]. For this reason, the transfer of strength gains from the NHE to sprint performance remains far from assured [39].

Current literature does not clearly establish whether the NHE provides advantages over alternative posterior-chain strength-training strategies, particularly with respect to injury outcomes and the transfer of eccentric hamstring strength gains to sprint and jump performance. For this reason, the aim of the present study was to compare the effects of a single strength-training protocol applied using two distinct posterior-chain approaches in elite academy youth football players. Specifically, one approach incorporated the traditional NHE, while the other employed alternative posterior-chain exercises. A further objective was to describe and compare the occurrence of hamstring injuries during the intervention period under both training protocols. The authors hypothesized that the NHE would not provide superior effects compared to other posterior chain exercises. Specifically, (a) while greater eccentric strength improvements were expected in the NHE group, these gains would not translate into superior sprint and jump performance, and (b) the incidence of HSIs would not differ significantly between intervention groups.

## 2. Materials and Methods

### 2.1. Participants

Twenty-three healthy elite male youth soccer players (mean age 13.7 ± 0.8 years) competing at the elite academy level within a professional club development program participated in this study. Players were randomly assigned to two groups using stratified randomization based on baseline eccentric hamstring strength, in accordance with established protocols [42]. Baseline strength was assessed during the pre-intervention testing session using the NHE performed on a NordBord device (Vald Performance, Newstead, Australia). Group allocation was performed after completion of baseline testing; assessors were not blinded during the allocation process. Goalkeepers were excluded from participation, as high-speed running, a primary mechanism underlying hamstring injuries, does not form a substantial part of their usual match demands. The athletes were distributed to either the Nordic Hamstring Exercise Group (NHEG; *n* = 11, height: 165.6 ± 8.0 cm, body mass: 54.9 ± 10.0 kg) or the Deadlift + Leg Curl Slides Group (D + LCSG; *n* = 12, height: 167.3 ± 6.1 cm, body mass: 54.0 ± 4.6 kg). All outfield playing positions were represented in both groups in a balanced manner. The NHEG included two fullbacks, two center backs, three midfielders, two wingers, and two strikers, while the D + LCSG included two fullbacks, two center backs, three midfielders, three wingers, and two strikers. All players maintained their habitual training routine, consisting of four weekly team practice sessions and one official match (~6 h in total). To be included in the study, participants were required to attend at least 85% of both resistance training and team practice sessions. The following participants were excluded from the study: four who were absent during training or testing, three who had sustained injuries prior to the intervention, and three who incurred an injury during the intervention period. All statistical analyses were conducted using data from the 23 players who met the inclusion criteria and completed both pre- and post-intervention testing. Players excluded prior to or during the intervention were not included in any analyses, and no missing data handling or imputation was required. Formal ethical approval was not required, as the study involved the retrospective analysis of fully anonymized data routinely collected as part of regular training, performance testing, and medical monitoring activities. Nevertheless, the study was conducted in accordance with the Declaration of Helsinki. The training intervention implemented in the study was fully integrated into the players’ normal training routine and did not involve procedures beyond standard practice or additional risk to participants. All data were anonymized prior to analysis, and no identifiable personal or sensitive information was used. Written informed consent was obtained from all participants and/or their legal guardians before data collection. Participation in the study was voluntary, and players were informed that declining participation or withdrawing at any time would not result in any negative consequences regarding team selection, playing time, or access to training and medical support. The coaching and performance staff were not involved in the consent process, and participation decisions were handled independently to minimize any potential perception of coercion.

### 2.2. Procedure

During the in-season period, both groups completed a comparable 9-week training program consisting of two weekly resistance training sessions in addition to regular on-field soccer practice (see Table 1). All resistance sessions were conducted in the gym under the supervision of at least two experienced performance coaches, who ensured correct exercise execution. The program was structured as a circuit, with recovery periods of 90–120 s between exercises. The first weekly session, aimed at strength development, was scheduled four days before match day (MD-4) immediately after soccer training and lasted approximately 30–40 min. The second session, focused on power development, was scheduled two days before match day (MD-2) before soccer training, with a duration of 25–30 min. In both training sessions, exercise intensity was autoregulated, with participants adjusting loads according to the prescribed number of repetitions and their perception of form, following the principle described by previous authors [43]. Where applicable, players were instructed to select a load that allowed them to complete the prescribed sets and repetitions while maintaining approximately two repetitions in reserve. This approach has demonstrated high effectiveness due to an appropriate balance between the intended and actual training stimulus [43]. The main difference between groups was the hamstring-targeted exercise performed during the strength session. The NHEG performed two sets of six repetitions of the NHE, while the D + LCSG alternated weekly between the Romanian Deadlift (2 × 6 rep at 80% 1RM) and the Leg Curl Slide (2 × 6 rep using body weight). All players were familiar with this type of training and with the specific exercises prior to the intervention. The same training structure had previously been applied in our earlier work, which followed an identical protocol but without the specific comparison between the NHE and alternative hamstring exercises, thereby supporting the consistency and reliability of the present design [44].

### 2.3. Testing Protocol

Several tests were conducted before and after the 9-week intervention period. Testing was performed over two consecutive days, separated by 24 h, to maintain regular training schedules and minimize fatigue [44]. Participants were instructed to avoid any physical activity for 48 h prior to testing. On Day 1, the testing sequence consisted of a jump test followed by the sprint test, whereas on Day 2, eccentric hamstring strength was assessed using the NordBord system. Each testing session began with a standardized 15 min warm-up including low-intensity running, mobility drills, and test-specific preparatory exercises.

### 2.4. Countermovement Jump Test (CMJ)

Jump ability was assessed using three CMJ trials on a force platform (SmartJump, Vald Performance, Newstead, Australia). Participants kept their hands on their hips throughout the movement, with countermovement depth self-selected to ensure upright landings with knee flexion, in accordance with established protocols [45], and any invalid trials were repeated. Jumps were separated by 45 s of passive recovery, and the best performance was retained for analysis.

### 2.5. Linear Sprint Test

Sprint performance was assessed over 30 m using photocell timing gates (SmartSpeed, Vald Performance, Newstead, Australia), with split times recorded at 10 m. Participants started from a standing position 50 cm behind the first gate, consistent with previous studies [46], and initiated the sprint at their own discretion to eliminate reaction time bias [47]. Two maximal sprints were performed, separated by 2 min of passive recovery, and the fastest time, recorded to the nearest 0.01 s, was retained for analysis.

### 2.6. NordBord Test

Eccentric hamstring strength was assessed using the NHE on a NordBord device (Vald Performance, Newstead, Australia). Players performed the NHE in a kneeling position with their feet secured to the hooks, which allowed measurement of the vertical force exerted and simultaneous recording of force values for both legs. After a standardized warm-up (3 × 3 repetitions at approximately 70%, 80%, and 90% of perceived maximal effort), they performed three maximal-effort repetitions following established protocols [48]. Hands remained crossed over the chest, and participants resisted the forward fall while maintaining a straight line from shoulders to knees. Knee position was recorded to ensure reproducibility, and the highest bilateral average force value was used for analysis, consistent with previous articles [49,50].

### 2.7. Injury Data Collection and External Load

During the entire intervention period, injury data were recorded by the team’s medical staff, who were not informed of players’ group allocation; however, only hamstring injuries sustained in training or matches were included in the present analysis. A recordable hamstring injury was defined as any posterior thigh injury incurred during training or competition that resulted in absence from subsequent football activities [51]. Injury incidence was reported as absolute counts and as rates per 1000 player-hours for matches and training [51]. Similarly, training and match exposure (official and friendly games) were quantified using GPS tracking data (Wimu Pro, Realtrack Systems, Almeria, Spain). The observation period lasted 12 weeks, including two additional weeks after the tests to monitor the occurrence of any further injuries. During these two additional weeks, the football players continued with their regular training routine.

### 2.8. Statistical Analysis

Data are presented as mean ± standard deviation (SD). Statistical analyses were performed using SPSS 2020 (Inc., Chicago, IL, USA). The Shapiro–Wilk test was first applied to confirm the normal distribution of the data. Within-group changes from pre- to post-training were evaluated using paired *t*-tests. Pretest values were included as covariates to analyze between-group changes from pre- to post-training. Data were further analyzed using a 2 (NHEG vs. D + LCSG) × 2 (pre vs. post) factorial ANOVA, with Bonferroni adjustments applied for post hoc comparisons. Statistical significance was set at *p* < 0.05. Effect sizes (ES) were calculated to quantify the magnitude of changes, and Cohen’s thresholds were interpreted as trivial (0.0–0.19), small (0.2–0.59), moderate (0.6–1.1), large (1.2–1.9), and very large (>2.0) [52].

## 3. Results

The responses of each group to the different variables examined during the intervention are presented in Table 2. NHEG showed a statistically significant increase in bilateral eccentric hamstring strength during the NHE *(p* ≤ 0.05; small ES). The remaining variables assessed (CMJ, 10 m sprint and 30 m sprint) showed no statistically significant differences in the post-test compared to the pre-test values (*p* > 0.05; from trivial to small ES). Similarly, the D + LCS group showed no significant differences between the two assessments for any of the variables analyzed (*p* > 0.05, from trivial to small ES). 

The between-group comparison is presented in Table 3. The results indicated that the intervention did not produce significant differences between groups in any of the performance variables (all *p* > 0.05).

Over the 12-week monitoring period, the NHE group accumulated a total exposure of 831.6 h (range from 814.0 to 849.2), while the D + LCS group accumulated 922.8 h (range from 909.6 to 936.0). Mean training and match exposures were 652.3 h (range from 636.9 to 667.7) and 179.3 h (range from 175.9 to 183.7), respectively, for the NHE group, and 724.8 h (range from 714 to 735.6) and 198.0 h (range from 194.4 to 201.6), respectively, for the D + LCS group (Table 4). Over the course of the intervention period, one hamstring injury was documented. This injury occurred during a training session and involved a player from the NHE group (Table 4); it was classified as a 2B strain of the right semimembranosus muscle at the myotendinous junction [53], resulting in 18 days of time loss.

Overall, the primary outcome was a small increase in eccentric hamstring strength in the NHE group, with no meaningful changes in sprint or jump performance in either group. No between-group differences were observed for performance outcomes, and hamstring injury incidence remained low across both training strategies, with only one injury in the NHE group.

## 4. Discussion

This study examined the effects of a 9-week posterior-chain strength-training program using two different approaches (the NHE and alternative posterior-chain exercises) in elite youth football players. A key finding was that inclusion of the NHE led to a modest, exercise-specific, increase in bilateral NHE strength; however, this improvement did not translate into enhanced sprint or jump performance. Both groups exhibited similar adaptations in response to the training program, with no significant between-group differences in any performance variable. Both programs reported a low incidence of hamstring injuries per 1000 h of exposure, with no statistically significant protective effect associated with the use of the NHE. These findings should be interpreted in the context of a relatively short, late-season intervention period, which may have limited the magnitude of observable adaptations and between-group differences.

The present findings are consistent with previous evidence supporting the effectiveness of the NHE in increasing eccentric knee flexor strength during NHE [24]. In the current study, the 6.3% improvement observed among football players performing the NHE was relatively lower than the increases previously reported in the literature, which ranged from 19% in adult footballers [34] to 11–17% in youth players [44]. These discrepancies may be explained by differences in training status, maturity level, and neuromuscular adaptation potential among the samples analyzed [54]. In contrast, players who performed Romanian Deadlifts and Leg Curl Slides did not exhibit statistically significant improvements in eccentric knee flexor strength assessed using the NHE. However, a small positive effect (ES = 0.26) was observed, indicating a limited yet potentially meaningful trend toward improvement. Interestingly, similar protocols in previous studies conducted earlier in the season have reported greater increases in eccentric hamstring strength [44]. This discrepancy in efficacy may be attributed to the higher biomechanical specificity of the NHE relative to the NordBord test, meaning that strength gains tend to be greatest when the training exercise closely resembles the testing movement. In practical terms, adaptations are more likely to transfer between tasks that share similar joint actions, muscle involvement, and contraction characteristics [24]. For example, previous research had shown that strength gains following an NHE protocol were smaller [55], or even absent [56], when eccentric knee flexor strength was assessed using isokinetic testing rather than with the NHE itself. Unfortunately, no additional hamstring strength assessments more closely resembling the Romanian deadlift or leg curl slide were included in the present study, which precluded verification of whether this rationale also applied in this case.

The results of this study also showed that, regardless of the exercises performed, the training protocol did not lead to improvements in either jumping or sprinting ability among the players. This lack of performance improvement is likely explained by a combination of contextual and methodological factors rather than by the ineffectiveness of the exercises themselves. Several factors may help explain these findings. First, conducting the intervention at the end of the season may have contributed to a potential ceiling effect, as suggested by previous literature [42], although no direct load or fatigue markers were analyzed in the present study. Secondly, the relatively low training volume implemented in this study (2 × 6 repetitions per week) may have been insufficient to elicit meaningful adaptations capable of improving the performance tests employed. Although the literature remains somewhat controversial regarding optimal training volume, some studies reported that higher NHE volumes were more effective for enhancing eccentric hamstring strength [57], while others indicated that low-volume protocols could produce comparable improvements to higher-volume approaches in elite youth and adult players [42,58,59]. It is still uncertain whether substantially increasing NHE volume during the in-season period would necessarily result in superior adaptations [59]. This uncertainty is particularly relevant given the potential for increased fatigue, time constraints, and delayed-onset muscle soreness, factors often cited as barriers to consistent exercise adherence [60]. Furthermore, as previously discussed, the degree of transferability between the training exercise and the test used to assess performance is a key determinant of observable improvements. The limited transfer between the NHE, Romanian Deadlift, and Leg Curl Slide, and the movement patterns involved in sprinting and the CMJ, may explain the absence of performance gains after the 9-week training period. In agreement with these findings, previous studies have also reported weak or absent associations between NHE-induced strength gains and sprint performance outcome [39,40,41]. The limited biomechanical and neuromuscular similarity between the slow, knee-dominant NHE and the high-velocity, hip-dominant demands of sprinting [35,36] likely accounts for the lack of improvement observed in the sprint test. Similarly, exercises such as the Romanian Deadlift and Leg Curl Slide, while effective for strengthening the posterior chain, have shown little to no transfer to sprint or jump performance in previous research [44], likely due to their limited movement specificity and contraction velocity relative to these explosive tasks. Based on these considerations and previous literature, it could be suggested that to improve sprint performance, strength training protocols for young players should include exercises with greater transferability, such as resisted sprint training [61], hip thrusts [62], exercises involving faster contraction modes [63], or simply a greater volume of sprint-specific training [64].

Similarly, the absence of improvements in CMJ performance could be explained by a combination of factors rather than by exercise specificity alone. Although both training protocols included multi-joint movements such as the back squat, which share certain mechanical characteristics with vertical jumping, the overall training stimulus may not have been sufficient to elicit meaningful adaptations in explosive performance [65]. Although the NHE group showed significant improvements in eccentric hamstring strength, previous research has reported weak associations between hamstring-oriented strength gains and jump performance outcomes [58]. The D + LCS group did not report any increases in hamstring strength. Consequently, the adaptations induced by these protocols may have had limited transfer to CMJ performance due to the different neuromuscular and biomechanical demands involved [66]. Likewise, although some plyometric and power-oriented exercises were included in the program, their total volume may not have provided an adequate stimulus to enhance rapid force production or stretch–shortening cycle efficiency, while the relatively low contraction velocity typical of traditional strength exercises may have further constrained improvements in vertical jump ability [63]. Although a previous study using a similar training protocol reported significant improvements in CMJ performance, it is important to note that this intervention was conducted earlier in the season, when players were likely experiencing lower cumulative fatigue and greater responsiveness to training stimuli. Previous authors have also reported that CMJ-based improvements tend to be smaller in the later stages of the season compared with the beginning, suggesting that seasonal timing and accumulated load may partially explain the lack of CMJ gains observed in the present study [67]. From a practical standpoint, to optimize CMJ adaptations during the later stages of the season, practitioners could consider slightly reducing the overall training load to alleviate fatigue, and incorporating higher volumes of explosive, high-velocity, or plyometric exercises.

With regard to injury outcomes, only one hamstring injury was recorded during the study period, occurring in a player from the NHE group. Based on the accumulated exposure (922.8 h for the NHEG vs. 831.6 h for the D + LCSG), this corresponds to an incidence rate of approximately 1.2 injuries per 1000 exposure hours in the NHEG and 0.0 injuries per 1000 exposure hours in the D + LCSG. The incidence observed in the NHEG was slightly higher than that reported in elite youth football players (~0.58 per 1000 h) [68] but remains within the broader range described in adult soccer populations (0.3–1.9 per 1000 h) [69]. Conversely, the absence of injuries in the D + LCSG resulted in an incidence below the values typically observed in the literature. However, these between-group differences must be interpreted cautiously due to the very small sample size (*n* = 12), which substantially limits the ability to detect meaningful differences. Overall, the low number of injuries suggests that the preventive framework implemented in this study may have contributed to limiting injury occurrence, regardless of the specific exercise performed. However, this finding should be interpreted cautiously given the very low number of injury events. Although meta-analyses have shown large reductions in HSI incidence following NHE implementation [22,23,24], these findings should be interpreted with caution due to methodological limitations, such as varied control conditions and the lack of studies in elite youth players. The present findings indicate that, when implemented within a comprehensive injury-prevention framework, different posterior-chain exercises were associated with similarly low hamstring injury incidence during the observation period [70]. In this context, the inclusion of the NHE resulted in exercise-specific strength gains but did not lead to a lower injury incidence compared with alternative posterior-chain exercises. These findings support the value of the NHE as part of a broader preventive strategy, while suggesting that it should not be regarded as a unique or “magic bullet” solution for hamstring injury prevention, as comparable outcomes were observed with other exercise approaches. Longer-term, multi-season investigations with larger samples are warranted to clarify the role of the NHE relative to alternative posterior-chain exercises in reducing hamstring injury risk in this population [60]. Overall, the NHE should be regarded as a complement to hip-dominant and sprint-based exercises rather than a standalone prevention tool. From a coaching perspective, these findings collectively indicate that while eccentric hamstring strength development remains an important component of youth football training, its transfer to improvements in sprint and jump performance may be limited. For this reason, practitioners should therefore combine posterior-chain strength work with high-velocity and task-specific exercises, such as sprinting, plyometric, and other explosive exercises that better reflect the mechanical and neuromuscular demands of match actions.

Several limitations should be considered when interpreting these findings. First, the small sample size and the very low number of hamstring injury events substantially limit the statistical power of the study, particularly with respect to injury incidence and between-group comparisons. Consequently, statistical inferences should be interpreted judiciously, and conclusions regarding injury prevention must remain cautious. For this reason, future studies with larger sample sizes should be conducted to further investigate this area. Second, the relatively short duration of the intervention likely constrained the magnitude of observable adaptations, particularly for performance outcomes and cumulative injury-prevention effects. Future interventions with longer intervention periods or multi-season investigations are required to more robustly evaluate performance transfer and injury-related outcomes. Third, eccentric hamstring strength was assessed using a test specific to the NHE, which favors the detection of task-specific adaptations. The absence of alternative strength assessments more closely reflecting the Romanian deadlift or leg curl slide limits the ability to compare exercise-specific strength adaptations between groups. In light of this, it should be examined whether more exercise-specific tests might have yielded different results. Fourth, although the intervention was conducted during the later stages of the competitive season, no direct load or fatigue markers were analyzed. Therefore, the proposed end-of-season ceiling effect should be regarded as a plausible, literature-supported interpretation, rather than a conclusion derived from direct monitoring data. Similarly, biological maturation was not directly assessed, which may have contributed to inter-individual variability in training responses. For this reason, future studies incorporating markers of fatigue and assessments of biological maturation are warranted.

## 5. Conclusions

In conclusion, the findings indicate that neither of the two implemented strength-training protocols produced improvements in jump or sprint performance in young soccer players by the end of the season, showing improvements only in bilateral eccentric hamstring strength assessed using the NHE, reflecting a task-specific adaptation, in the group that included the NHE in their routines. Over the study period, a low number of hamstring injuries was observed in both groups; therefore, injury-related findings should be interpreted cautiously. Within the limits of the present study, both protocols were associated with a low occurrence of hamstring injuries, and the inclusion of the NHE did not appear to confer additional benefits compared with alternative posterior-chain exercises. Accordingly, performance and medical staff may consider integrating the NHE, Romanian deadlifts, or leg curl slides into soccer training programs as part of a comprehensive injury-prevention framework, while including supplementary exercises specifically designed to enhance sprint and jump performance.

## Figures and Tables

**Table 1:** Content of the strength and power session.

		Sets	Reps	Intensity
**Strength training**	Nordic hamstring exercise or Leg curl slides	2	6	BW
	Barbell back squats	2	8	RIR 2
	Dynamic Copenhagen side plank	2	10 each side	BW
	Rear-step lunge	2	6 each side	RIR 2
	Rotational anti-extension press	2	10 each side	Elastic resistance
	Horizontal bench press	2	8	RIR 2
	Dumbbell single arm row	2	8 each side	RIR 2
**Power training**	Medball horizontal throw	2	3	4–6 kg
	Lateral bounds	2	3 each side	BW
	Jump-to-box	2	5	60–70 cm height
	Drop jumps	2	4	30–40 cm height
	Resisted accelerations	2	5	Elastic resistance
	Resisted lateral shuffles	2	3 each side	Elastic resistance

BW: bodyweight; RIR: Repetitions in reserve.

**Table 2:** Changes in different performance variables after the training intervention (mean ± SD).

	Variables	Pre Intervention	Post Intervention	Change Mean (%)	ES (95%CI)	*p* Value
D + LCS Group (*n* = 12)	NHEes (N)	270.5 ± 40.9	281.4 ± 39.6	4.2 ± 6.9	0.24 (−0.15; 0.63)	0.20
	CMJ (cm)	40.4 ± 3.7	39.7 ± 3.8	−1.9 ± 7.2	−0.19 (0.85; 0.47)	0.54
	Sprint 10 m (s)	1.77 ± 0.07	1.78 ± 0.06	0.4 ± 0.7	0.08 (−0.08; 0.24)	0.27
	Sprint 30 m (s)	4.30 ± 0.17	4.31 ± 0.18	0.1 ± 0.7	0.02 (−0.13; 0.17)	0.91
NHE Group (*n* = 11)	NHEes (N)	274.5 ± 52.4	290.0 ± 44.9	6.3 ± 5.8	0.27 (0.02; 0.53)	0.04
	CMJ (cm)	38.2 ± 4.9	38.2 ± 3.3	0.3 ± 9.0	−0.01 (−0.62; 0.60)	0.98
	Sprint 10 m (s)	1.83 ± 0.07	1.84 ± 0.06	0.3 ± 2.4	0.08 (−0.49; 0.65)	0.77
	Sprint 30 m (s)	4.46 ± 0.17	4.45 ± 0.18	−0.4 ± 1.9	−0.09 (−0.53; 0.35)	0.67

ES: Effect size; NHEes: eccentric strength during Nordic hamstring exercise; and CMJ: counter movement jump.

**Table 3:** Comparison of performance variables after training protocol across groups.

	Variables	Difference in Means	95% CI [LB, UB]	*p* Value
D + LCS Group (*n* = 12) vs. NHE Group (*n* = 11)	NHEes (N)	0.1	[−0.3; 0.5]	0.65
	CMJ (cm)	0.2	[−0.7; 1.0]	0.71
	Sprint 10 m (s)	0.0	[−0.6; 0.5]	0.97
	Sprint 30 m (s)	−0.1	[−0.5; 0.3]	0.62

LB: lower bound; UB: upper bound; NHEes: eccentric strength during Nordic hamstring exercise; and CMJ: counter movement jump.

**Table 4:** Comparison of injury data collection between groups.

	D + LCS Group (*n* = 12)	NHE Group (*n* = 11)
Total Exposure (h)	922.8 ± 13.2	831.6 ± 17.6
Trainings exposure (h)	724.8 ± 10.8	652.3 ± 15.4
Matches exposure (h)	198 ± 3.6	179.3 ± 4.4
Hamstring injuries	0	1
Total Hamstring injury rate/1000 h	0.0	1.20

## Data Availability

The data presented in this study are available on request from the corresponding author.
